# The use of pre-operative virtual reality to reduce anxiety in women undergoing gynecological surgeries: a prospective cohort study

**DOI:** 10.1186/s12871-020-01177-6

**Published:** 2020-10-09

**Authors:** Jason Ju In Chan, Cheng Teng Yeam, Hwei Min Kee, Chin Wen Tan, Rehena Sultana, Alex Tiong Heng Sia, Ban Leong Sng

**Affiliations:** 1 Department of Women’s Anesthesia, KK Women’s and Children’s Hospital, 100 Bukit Timah Road Singapore, Singapore City, 229899 Singapore; 2 Duke-NUS Medical School, 8 College Road Singapore, Singapore City, 169857 Singapore; 3 Division of Nursing, KK Women’s and Children’s Hospital, Singapore City, Singapore; 4 Centre for Quantitative Medicine, Duke-NUS Medical School, Singapore City, Singapore

**Keywords:** Virtual reality, Preoperative anxiety, Patient satisfaction

## Abstract

**Background:**

Pre-operative anxiety is common and is associated with negative surgical outcomes. Virtual reality (VR) is a promising new technology that offers opportunities to modulate patient experience and cognition and has been shown to be associated with lower levels of anxiety. In this study, we investigated changes in pre-operative anxiety levels before and after using VR in patients undergoing minor gynecological surgery.

**Methods:**

Patients who underwent elective minor gynecological surgeries in KK Women’s and Children’s hospital, Singapore were recruited. The VR intervention consisted of 10-min exposure via a headset loaded with sceneries, background meditation music and breathing exercises. For the primary outcome of pre-operative anxiety, patients were assessed at pre- and post-intervention using the Hospital Anxiety and Depression Scale (HADS). Secondary outcomes of self-reported satisfaction scores and EuroQol 5-dimension 3-level (EQ-5D-3L) were also collected.

**Results:**

Data analysis from 108 patients revealed that HADS anxiety scores were significantly reduced from 7.2 ± 3.3 pre-intervention to 4.6 ± 3.0 post-intervention (*p* < 0.0001). Furthermore, HADS depression scores were significantly reduced from 4.7 ± 3.3 pre-intervention to 2.9 ± 2.5 post-intervention (*p* < 0.0001). Eighty-two percent of the patients self-reported VR intervention as ‘Good’ or ‘Excellent’. EQ-5D-3L showed significant changes in dimensions of ‘usual activities’ (*p* = 0.025), ‘pain/discomfort’ (*p* = 0.008) and ‘anxiety/ depression’ (*p* < 0.0001).

**Conclusions:**

For patients undergoing minor gynecological procedures, the VR intervention brought about a significant reduction in pre-operative anxiety. This finding may be clinically important to benefit patients with high pre-operative anxiety without the use of anxiolytics.

**Trial registration:**

This study was registered on clinicaltrials.gov registry (NCT03685422) on 26 Sep 2018.

## Background

Anxiety can be defined as emotions of fear, tension or unease and is often encountered before surgery [1, 2]. Pre-operative anxiety has been shown to be correlated with acute and chronic post-surgical pain, increased use of post-operative analgesia and post-operative nausea and vomiting [3–5]. It also has significant impact on recovery, including longer post-operative hospital stay and even cognitive and behavioral ramifications [2–5]. Furthermore, women often experiencing higher levels of pre-operative anxiety compared to men [2, 6, 7]. While pharmacological interventions for pre-operative anxiety are available, reservations such as safety profile and cost often hinder physicians to fully utilize them. Therefore, non-pharmacological methods such as music and Virtual Reality (VR) are gradually growing in popularity to improve the overall patient surgical experience [8–12].

The use of VR therapy in various clinical settings is well documented, such as physical rehabilitation, pain distraction, overcoming phobias, anxiety disorders, and post-traumatic stress disorder (PTSD) [13, 14]. It is reported that VR therapy results in significantly reduced anxiety, persistent pain intensity, faster wound healing, and improved neurorehabilitation outcomes in patients with burns and complex regional pain syndrome [15, 16]. The technology usually consists of an audio system (earphones or headphones), a visual system (head-mounted displays) and an integrated set up (motion tracking systems). By providing multiple stimuli to the human senses, VR systems are able to allow the user an immersive experience and presence in the virtual world [17–19].

In the gynecological population, limited evidence has been reported on the use of VR therapy for postoperative care and management. In a non-randomized controlled study recruiting patients undergoing colposcopy (cervical examination), Vasquez et al. showed that patients assigned to VR group reported reduced pain scores post-VR intervention [20]. Another prospective randomized controlled trial in an outpatient hysteroscopy setting showed that the use of VR during the procedure resulted in significantly decreased average pain score and anxiety when compared to controls [21]. Nevertheless, there are limited studies conducted in a gynecological population, and no formal sample size calculations were performed to study the expected clinical effect size related to pre-operative anxiety.

In view of the potential clinical benefits of VR, our study aimed to assess pre-operative anxiety (primary outcome) and self-reported satisfaction of VR and health state (secondary outcomes) in women undergoing minor gynecological procedures.

## Methods

This prospective cohort study was conducted between March 2019 and January 2020 at KK Women’s and Children’s Hospital, Singapore. The study protocol adhered to the Strengthening the Reporting of Observational studies in Epidemiology (STROBE) guidelines and was approved by the SingHealth Centralized Institutional Review Board, Singapore (SingHealth CIRB Ref: 2018/2200), and registered on Clinicaltrials.gov (ID: NCT03685422).

### Inclusion and exclusion criteria

Women aged 21–70 years old, American Society of Anesthesiologist (ASA) physical status I or II, with no visual or mental impairment and undergoing gynecological surgery were included in this study. Patients with severe motion sickness, significant respiratory disease or obstructive sleep apnea, oncological gynecology and obstetrics patients were excluded. Women who were unable to communicate in English or unable to understand the administered questionnaires were also excluded from this study.

### Psychometric assessment tools used

The State-Trait Anxiety Inventory (STAI) designed by Spielberger et al. has been used extensively in research and clinical settings [22]. It has been used to measure the presence and severity of current symptoms of anxiety and a generalized propensity to be anxious. The tool consists of 40 items, 20 allocated each to state-anxiety and trait-anxiety. All items are rated on a 4-point Likert scale (e.g. from “Not at all” to “Very much so”; or from “Almost never” to “Almost always”). Test-retest reliability coefficients on initial development ranged from 0.31 to 0.86, with intervals ranging from 1 h to 104 days [22].

The Hospital Anxiety and Depression Scale (HADS) is commonly used to assess the patients’ level of anxiety and depression during their hospitalization and is preferentially used as an indicator for global psychological distress [23]. Each item on the questionnaire is scored from 0 to 3, thus a patient may have a total score from 0 to 21 for the anxiety and depression subscales, respectively. A score of 0–7 indicates normal level of anxiety/depression while 8–10 indicates borderline abnormal and 11–21 indicates abnormal. Validity of the HADS was deemed “good” to “very good”, with comparable sensitivity and specificity of longer scales including the STAI and the Symptom Checklist-90 anxiety scales [24]. HADS has been validated in gynecological populations undergoing procedures, achieving good levels of internal consistency with Cronbach’s α of 0.78 and 0.84 for anxiety and depression subscales, respectively, and 0.88 for the whole instrument [25]. As compared with conventional instruments that measure anxiety (e.g. STAI), the shorter HADS provides increased convenience for patients and allows for multiple measurements at different time points pre and post intervention.

The EuroQol 5-dimension 3-level (EQ-5D-3L) questionnaire [26] is one of the most widely used instruments for measuring health-related quality of life. It consists of a descriptive system on health state comprising five dimensions (5D) with three levels (3 L) of self-reporting in each dimension: mobility, self-care, usual activities, pain/discomfort, and anxiety/depression; each dimension ranging from 1 to 3 to reflect level of impairment of “with no problem”, “with some problems” or “with severe problems”. The evaluation component involves a visual analog scale (VAS), asking to mark health state on the day of interview on a 20 cm vertical scale with end point of 0 and 100. Zero corresponds to “the worst health you can imagine” and hundred corresponds to “the best health you can imagine”. For measuring patient satisfaction with regards to the VR intervention, a self-reported 4-point Likert scale with the following items: “Poor”, “Fair”, “Good” and “Excellent” was used. Pain score at rest was scored using a 0–10 Numerical Rating Scale (NRS).

### Patient recruitment

Patients presenting to the day surgery service for a variety of minor gynecological procedures were initially screened by study investigators using the operative room surgical listing schedule. The investigators then evaluated the patient’s medical records to determine her eligibility. Patients meeting inclusion criteria were approached in a pre-operative holding area. Risks and benefits of the study were explained, and informed consent was obtained. No patient remuneration was provided in this study.

**Fig. 1:**
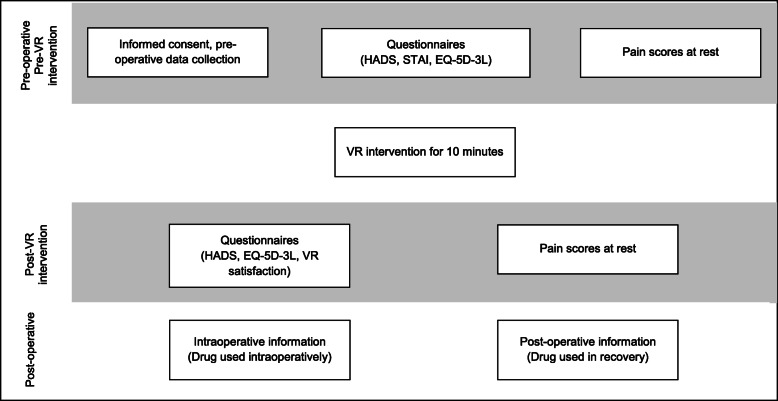
Study flowchart

**Fig. 2:**
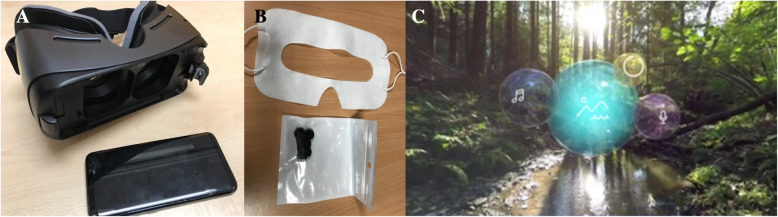
The setting of VR intervention. **a** The Samsung 8 smartphone for attaching onto a Samsung Gear VR 3; **b** Disposable sanitary covers and earbuds were provided for each use; and **c** A screenshot of menus of Relax VR. Used with permission from Relax VR [27]

Pre-VR intervention assessments included demographic data, pain score and psychometric questionnaires (STAI, HADS and EQ-5D-3L; Fig. 1). Patients were then given a Samsung Gear VR3 (Samsung Co. Ltd) headset and audio earpieces, fitted with a Samsung 8 smartphone (Fig. 2a) running ‘Relax VR’ program (Fig. 2c) [27]. Disposable sanitary covers and earbuds were provided, that were discarded and replaced between each user (Fig. 2b). Patients were given eleven immersive scenarios to choose from, and the experience was integrated with background meditation music and breathing exercises. The eleven scenarios included sceneries from a tropical beach in the Philippines, a rice terrace in the Philippines, wine glass bay beach in Australia, the Twelve Apostles in Australia, Fern Bern in New Zealand, a forest creek in Germany, a daisy garden in Germany, the Grand Canyon in the (United States of America) USA, watching northern lights in the USA, floating in the sky in clouds and being on the moon in outer space. The VR intervention was conducted with patients lying in bed in the fowler’s position with knees straight, in a quiet pre-operative waiting area. Patients were able to move their body freely in bed while on the headsets and were also instructed to discontinue the VR intervention if they experienced any side effects such as motion sickness or dizziness.

After the VR intervention, pain score, satisfaction score and psychometric assessments (HADS, EQ-5D-3L) were performed and collected. The VR intervention lasted for 10 min, with pre- and post-intervention surveys all done 1–2 h before surgery. The patients subsequently underwent their intended surgical procedure under general anesthesia. Intra- and post-operative care provided adhered to standard hospital protocol. Data on intra- and post-operative analgesic use was also collected. Patients were all admitted to the day surgery unit in the hospital post-operatively before being further assessed to be discharged or for longer hospital stay. Data on analgesic use and pain score were also collected post-operatively in the recovery area.

The primary outcome for this study was the change in pre-operative anxiety as quantified by the HADS scale. The HADS anxiety scores pre- and post-VR were compared for data analysis. For secondary outcomes, EQ-5D-3L and patient satisfaction of the VR intervention were targeted and used for data analysis of the patient’s health state and anxiety levels.

### Sample size calculation and statistical analysis

Tan et al. [28] reported difference in mean (standard deviation (SD)) HADs anxiety between pre- and post-intervention in music experiences as 4.61 (4.08). The calculated sample size of 70 was based on the following assumptions: considering a conservative mean (SD) HADS difference of 2.0 (8.0), level of significance as 5% and power as 90%. After adjusting for 40% loss to follow up, ineligibility and withdrawal, a recruitment goal of 110 patients was targeted.

Categorical and continuous variables were summarized as frequency (proportion) and mean ± SD respectively. Difference between pre- and post-VR experiences were compared using paired t – test and McNemar test for paired continuous and paired categorical data respectively. *P*-value < 0.05 was considered as statistical significance and all the tests were two – sided. Analyses were done using SAS version 9.4 software (SAS Institute; Cary, North Carolina, USA).

## Results

A total of 110 patients aged 24–59 years old were recruited but only 108 patients’ data were analyzed as two patients withdrew prior to the intervention. Table 1 shows the demographic data for the patients. Majority of the patients were of Chinese ethnicity (70.37%), ASA 1 status (72.22%) and underwent dilatation, curettage and hysteroscopy (82.41%) (Table 1). No adverse events were reported during and post VR intervention, and we observed no motion sickness nor dizziness in the recruited patients. Pre-operative HADS scores compared between types of surgery showed no significant difference (*p* = 0.4879). Eighty-eight patients (81.5%) were discharged on the same day of their surgery, whereas the rest (*n* = 20 or 18.5%) were hospitalized overnight.

**Table 1:** Patient Demographics

Characteristics	Mean ± SD/ ***n*** (%)
Age (years)	43.56 ± 6.68
Race	
Chinese	76 (70.37)
Malay	15 (13.89)
Indian	4 (3.70)
Others	13 (12.04)
ASA status	
Class 1	78 (72.22)
Class 2	30 (27.78)
Weight (kg)	64.60 ± 12.54
Height (cm)	158.56 ± 5.97
Duration of Surgery (min)	26.43 ± 41.86
Type of surgery	
Dilatation and Curettage, Hysteroscopy	89 (82.41)
Others	19 (17.59)

Values are represented as mean ± standard deviation (SD) or number (%)

*ASA* American Society of Anesthesiologists

Pre- and post-VR psychological outcomes are displayed in Table 2. Importantly, for our primary outcome, there were statistically significant reduction in anxiety and depression using HADS (*p* < 0.0001). Furthermore, for our secondary outcome, anxiety/depression (*p* < 0.0001), self-reported perception of pain and discomfort (*p* = 0.0073) and perceived health states (*p* < 0.0001) in EQ-5D-3L also showed statistically significant improvements post-VR intervention pre-operatively. There was no significant association between STAI and change in HADS anxiety scores (*p* = 0.6352).

**Table 2:** Pre-Virtual Reality and Post-Virtual Reality psychological outcomes

Variables	Pre-VR	Post-VR	***P*** value
STAI S-anxiety score	39.59 ± 11.14	–	–
STAI T-anxiety score	40.10 ± 9.07	–	–
STAI total score	79.69 ± 18.78	–	–
HADS score			
Anxiety	7.23 ± 3.27	4.62 ± 3.03	< 0.0001
Depression	4.12 ± 3.34	2.92 ± 2.51	< 0.0001
EQ-5D-3L dimensions anxiety/depression			< 0.0001
Not anxious/depressed	62 (57.41)	90 (83.33)	
Having anxious/depressed	46 (42.59)	18 (16.67)	
EQ-5D-3L dimensions Pain/Discomfort			0.0073
No pain/discomfort	72 (66.67)	84 (77.78)	
Having pain/discomfort	36 (33.33)	24 (22.22)	
EQ-5D-3L VAS health state	71.57 ± 17.75	76.05 ± 15.07	< 0.0001

Values are represented as mean ± standard deviation (SD) or number (%)

*HADS* Hospital Anxiety and Depression Scale, *STAI* State-Trait Anxiety Inventory, *VAS* Visual analog scale, *VR* Virtual reality

Table 3 displays values of EQ-5D-3L for all five dimensions and three levels in detail, with the number of patients reporting each level within each dimension pre-VR and post-VR intervention. There was a statistically significant difference on EQ-5D-3L VAS health state (mean ± SD) of pre- and post-VR intervention (71.57 ± 17.75 vs 76.05 ± 15.07; *p* < 0.001). Table 4 shows the pain and satisfaction scores collected. Notably, pain scores collected pre- and post-VR intervention did not reveal any significantly changes (*p* = 0.2178). Intra- and post-operative pharmacological information, including type, dosage and route of analgesia, are displayed in Table 4. Significantly, for the secondary outcome of patient satisfaction of the VR intervention, 82.41% of the participants rated the experience as ‘Good’ or ‘Excellent’ (Table 4).

**Table 3:** EQ-5D-3L individual dimensions

Dimension	Level 1		Level 2		Level 3		***P*** value
	Pre-VR	Post-VR	Pre-VR	Post-VR	Pre-VR	Post-VR	
Mobility	106 (98.15)	107 (99.07)	1 (0.93)	1 (0.93)	1 (0.93)	0 (0.00)	0.3170
Self-care	108 (100.00)	108 (100.00)	0 (0.00)	0 (0.00)	0 (0.00)	0 (0.00)	–
Usual Activities	102 (94.44)	107 (99.07)	6 (5.56)	1 (0.93)	0 (0.00)	0 (0.00)	0.0250
Pain/ Discomfort	72 (66.67)	84 (77.78)	36 (33.33)	24 (22.22)	0 (0.00)	0 (0.00)	0.007
Anxiety/ Depression	62 (57.41)	90 (83.33)	46 (42.59)	18 (16.67)	0 (0.00)	0 (0.00)	< 0.0001

Values are represented as number (%)

**Table 4:** Pain and Satisfaction Scores

Characteristics	Mean ± SD/ ***n*** (%)
Pre-operative pain score pre-VR	0.44 ± 1.24
Pre-operative pain score post-VR	0.60 ± 1.21
Patient satisfaction on VR experience	
Excellent	35 (32.41)
Good	54 (50.00)
Fair	17 (15.74)
Poor	2 (1.85)
Maximum pain score post-operative in recovery	2.22 ± 2.41
Mean dose of Fentanyl used intra-operatively (mcg)	84.55 ± 19.49
Mean dose of Morphine used intra-operatively (mg)	5.79 ± 2.72
Paracetamol use intra-operatively	56 (51.85)
Duration of stay in the recovery unit (min)	64.50 ± 31.89
Fentanyl use in the recovery unit	16 (14.81)
Morphine use in the recovery unit	6 (5.56)
Paracetamol use in the recovery unit	9 (8.33)

Values are represented as mean ± standard deviation (SD) or number (%)

*VR* Virtual Reality

In terms of immersive VR scenario selection, majority of the participants (*n* = 24, 22.22%) selected Wine Glass Bay Beach, Australia, followed by Northern Lights, USA (*n* = 20, 18.51%), Tropical beach, Philippines (*n* = 19, 17.59%), Daisy Garden, Germany (*n* = 15, 13.89%), the Twelve Apostles, Australia (*n* = 9, 8.33%), Fern Bern, New Zealand (*n* = 6, 5.56%) and Forest Creek, Germany (*n* = 6, 5.56%).

## Discussion

By examining patients using VR intervention before undergoing minor gynecological procedures, we found that the use of a 10-min VR intervention resulted in a statistically significant reduction of pre-operative anxiety and depressive symptoms as measured using the HADS. While the pain scores collected pre- and post-VR intervention did not reveal any significantly changes, EQ-5D-3L measures further revealed that pre-operative self-reported perception of pain and discomfort and perceived health states were improved after VR intervention.

This study revealed that there is significant preoperative anxiety amongst the gynecological patients recruited, and is in congruence with other studies using HADS to measure changes in pre-operative anxiety for VR interventions in oncology patients [29, 30] and patients in intensive care [31]. Surgery is a daunting experience that comes with emotional vulnerabilities. These emotions are often intensified moments before surgery, causing overwhelming anxiety and even depressive moods [32]. Increased preoperative anxiety is associated with postponement or even cancellation of planned surgeries, increase in anesthetic requirements, prolonged hospital stay and poorer overall patient satisfaction [33, 34].

Patient-centric outcomes were investigated as part of our secondary outcomes in this study using the EQ-5D-3L. This provided other insights into patients’ health conditions, baseline functional status and quality of life. In this study, EQ-5D-3L assessment showed statistically significant improvement on self-reported pain/discomfort and anxiety/depression dimensions before gynecological surgery when VR was used. In addition, self-reported perception of ‘usual activities’ dimension also showed improvement post-VR. Furthermore, patients had overall positive self-reported satisfaction for the VR experience prior to their scheduled gynecological procedure.

In previous studies, patients who received VR treatment reported a reduction in pain and anxiety [16], faster wound healing [35], decreased chronic pain intensity [15] and other neuro-rehabilitation improvements [36]. These results largely corroborated with our findings, which showed reduction in anxiety. While the exact neurobiological mechanistic theory behind VR’s action remain unclear, it is generally suggested that VR acts as a distraction by rendering several possible mechanisms by: i) engaging different senses simultaneously and inducing a sense of presence in the virtual environment, thus diverting one’s attention from painful stimuli and other negative emotions such as stress and anxiety [37]; ii) employing attentional resources in immersive and interactive virtual environments to modulate ascending nociceptive stimuli and thus reduce pain experience [38]; iii) isolating the user both visually and acoustically from the actual environment to escape from the painful world cognitively [39]. VR could serve as a non-pharmacological intervention in clinical settings to modulate emotional affective, emotion-based cognitive and attentional processes [40]. Interestingly, although the mean pain scores pre- and post-VR intervention were not statistically significant, there was an improvement of self-reported perception in the dimension of ‘pain/discomfort’ in the EQ-5D-3L. The pain score changes could be attributed to pre-surgical administration of vaginal or oral prostaglandins for cervical softening.

### Study limitations

There were several limitations in our study. Firstly, the instruments used for assessment of anxiety were dependent on self-reported psychometric questionnaires. Although these psychometric tools have been validated in previous studies with similar target populations, there might be more suitable and sensitive measures of anxiety (e.g. STAI) and other psychometric measures (e.g. pain catastrophizing scale (PCS), perceived stress scale (PSS)) to reflect the effects of VR intervention on patients’ psychological profiles [41, 42]. Secondly, the patient population selected had to have the ability to read and understand English, which might limit the sociodemographic profiles of patients.

Thirdly, multiple factors unrelated to surgery could influence pre-operative anxiety. For example, we did not investigate interactions between study team investigator and the patient. Non-study team members and the surrounding environment may also affect the patient’s mood and anxiety. The effects of different scenarios on anxiety scores were also not studied due to an unequal distribution of scenarios that were chosen by patients. Finally, there was a lack of a control group to compare anxiety scores without VR intervention, making it difficult to assess the true effect of VR on pre-operative anxiety. Future randomized controlled trials are needed to validate our findings in this study.

## Conclusions

This study might have given some indication that VR relaxation technique could be a promising method for anxiety alleviation, improvement on pain perception and perceived health states during perioperative settings which could be extended for hospital use (rehabilitation, outpatient procedures, diagnostic scanning and perioperative period). This strategy may hence potentially increase patient satisfaction while providing non-pharmacological anxiolytic effects with minimal side effects.

## Data Availability

The datasets generated and analyzed in this work are available for anyone who wishes to access the data by contacting the corresponding author.

## Abbreviations

ASA: American Society of Anesthesiologists
HADS: Hospital Anxiety and Depression Scale
SD: Standard deviation
STAI: State-Trait Anxiety Inventory
STROBE: Strengthening the Reporting of Observational studies in Epidemiology
VAS: Visual analog scale
VR: Virtual reality

## References

[1] Johnston M (1980). Anxiety in surgical patients. Psychol Med.

[2] Carr E, Brockbank K, Allen S, Strike P (2006). Patterns and frequency of anxiety in women undergoing gynaecological surgery. J Clin Nurs.

[3] Suffeda A, Meissner W, Rosendahl J, Guntinas-Lichius O (2016). Influence of depression, catastrophizing, anxiety, and resilience on postoperative pain at the first day after otolaryngological surgery: a prospective single center cohort observational study. Medicine (Baltimore).

[4] Pinto PR, McIntyre T, Nogueira-Silva C, Almeida A, Araújo-Soares V (2012). Risk factors for persistent postsurgical pain in women undergoing hysterectomy due to benign causes: a prospective predictive study. J Pain.

[5] Gerbershagen HJ, Dagtekin O, Rothe T (2009). Risk factors for acute and chronic postoperative pain in patients with benign and malignant renal disease after nephrectomy. Eur J Pain.

[6] Moerman N, van Dam FS, Muller MJ, Oosting H (1996). The Amsterdam preoperative anxiety and information scale (APAIS). Anesth Analg.

[7] Matthias AT, Samarasekera DN (2012). Preoperative anxiety in surgical patients - experience of a single unit. Acta Anaesthesiol Taiwanica.

[8] Wang SM, Kulkarni L, Dolev J, Kain ZN. Music and preoperative anxiety: a randomized, controlled study. Anesth Analg 2002, 94(6):1489–1494, table of contents. 10.1097/00000539-200206000-00021 12032013

[9] Lechtzin N, Busse AM, Smith MT, Grossman S, Nesbit S, Diette GB (2010). A randomized trial of nature scenery and sounds versus urban scenery and sounds to reduce pain in adults undergoing bone marrow aspirate and biopsy. J Altern Complement Med.

[10] Dascal J, Reid M, IsHak WW (2017). Virtual reality and medical inpatients: a systematic review of randomized, Controlled Trials. Innov Clin Neurosci.

[11] Seiden SC, McMullan S, Sequera-Ramos L (2014). Tablet-based interactive distraction (TBID) vs oral midazolam to minimize perioperative anxiety in pediatric patients: a noninferiority randomized trial. Paediatr Anaesth.

[12] Tashjian VC, Mosadeghi S, Howard AR (2017). Virtual reality for Management of Pain in hospitalized patients: results of a controlled trial. JMIR Ment Health.

[13] Wiederhold BK, Wiederhold MD. Virtual reality therapy for anxiety disorders: advances in evalation and treatment. In: American Psychological Association; 2005.

[14] Wiederhold BK, Bouchard S. In Advances in virtual reality and anxiety disorders. In: Virtual Reality for posttraumatic stress disorder edn. Boston, MA.: Springer; 2014: 211–233.

[15] Sato K, Fukumori S, Matsusaki T (2010). Nonimmersive virtual reality mirror visual feedback therapy and its application for the treatment of complex regional pain syndrome: an open-label pilot study. Pain Med.

[16] Jeffs D, Dorman D, Brown S (2014). Effect of virtual reality on adolescent pain during burn wound care. J Burn Care Res.

[17] Lombard M, Ditton T. At the Heart of It All: The Concept of Presence. J Comput-Mediat Commun. 1997;3(2).

[18] Slater M (2018). Immersion and the illusion of presence in virtual reality. Br J Psychol.

[19] Wijnand Ijsselsteijn GR (2003). Being There: The experience of presence in mediated environments. Emerg Commun.

[20] Vasquez JMVVL, Wiederhold BK, Miller I, Wiederhold MD (2017). Virtual reality pain distraction during gynaecological surgery - a report of 44 cases. Surg Res Updates.

[21] Deo N, Khan KS, Mak J, et al. Virtual reality for acute pain in outpatient hysteroscopy: a randomised controlled trial. BJOG. 2020. 10.1111/1471-0528.16377 32575151

[22] Spielberger. Manual for the State-Trait Anxiety Inventory. rev. ed. 1983.

[23] SRP ZAS (1983). The hospital anxiety and depression scale. Acta Psychiatr Scand.

[24] Bjelland I, Dahl AA, Haug TT, Neckelmann D (2002). The validity of the hospital anxiety and depression scale. An updated literature review. J Psychosom Res.

[25] Watrowski R, Rohde A (2014). Validation of the polish version of the hospital anxiety and depression scale in three populations of gynecologic patients. Arch Med Sci.

[26] EuroQol G (1990). EuroQol--a new facility for the measurement of health-related quality of life. Health Policy.

[27] Relax VR [https://www.relaxvr.co/].

[28] Tan DJA, Polascik BA, Kee HM (2020). The effect of perioperative music listening on patient satisfaction, anxiety, and depression: a Quasiexperimental study. Anesthesiol Res Pract.

[29] Oyama HKM, Katsumata N, Akechi T, Ohsuga M (2000). Using the bedside wellness system during chemotherapy decreases fatigue and emesis in cancer patients. J Med Syst.

[30] Espinoza M, Banos RM, Garcia-Palacios A (2012). Promotion of emotional wellbeing in oncology inpatients using VR. Stud Health Technol Inform.

[31] Ong TL, Ruppert MM, Akbar M (2020). Improving the intensive care patient experience with virtual reality-a feasibility study. Crit Care Explor.

[32] Jawaid M, Mushtaq A, Mukhtar S, Khan Z (2007). Preoperative anxiety before elective surgery. Neurosciences (Riyadh).

[33] Tan DJ, Chan MM (2017). Do obstetric patients opt to undergo general Anaesthesia to avoid being conscious despite safer alternatives?. Ann Acad Med Singap.

[34] Jlala HA, French JL, Foxall GL, Hardman JG, Bedforth NM (2010). Effect of preoperative multimedia information on perioperative anxiety in patients undergoing procedures under regional anaesthesia. Br J Anaesth.

[35] Brown NJ, Kimble RM, Rodger S, Ware RS, Cuttle L (2014). Play and heal: randomized controlled trial of ditto™ intervention efficacy on improving re-epithelialization in pediatric burns. Burns.

[36] Wang M, Reid D (2011). Virtual reality in pediatric neurorehabilitation: attention deficit hyperactivity disorder, autism and cerebral palsy. Neuroepidemiology.

[37] Triberti S, Repetto C, Riva G (2014). Psychological factors influencing the effectiveness of virtual reality-based analgesia: a systematic review. Cyberpsychol Behav Soc Netw.

[38] Sharar SR, Alamdari A, Hoffer C, Hoffman HG, Jensen MP, Patterson DR (2016). Circumplex model of affect: a measure of pleasure and arousal during virtual reality distraction analgesia. Games Health J.

[39] Hoffman HG, Chambers GT, Meyer WJ (2011). Virtual reality as an adjunctive non-pharmacologic analgesic for acute burn pain during medical procedures. Ann Behav Med.

[40] Li A, Montano Z, Chen VJ, Gold JI (2011). Virtual reality and pain management: current trends and future directions. Pain Manag.

[41] Matheve T, Bogaerts K, Timmermans A (2020). Virtual reality distraction induces hypoalgesia in patients with chronic low back pain: a randomized controlled trial. J Neuroeng Rehabil.

[42] Wang TC, Sit CH, Tang TW, Tsai CL. Psychological and Physiological Responses in Patients with Generalized Anxiety Disorder: The Use of Acute Exercise and Virtual Reality Environment. Int J Environ Res Public Health. 2020;17(13). 10.3390/ijerph17134855 PMC7370051 32640554

